# Anterolateral thigh perforator flap made by customized 3D-printing fabrication of fixed positioning guide for oromaxillofacial reconstruction: a preliminary study

**DOI:** 10.4317/medoral.25558

**Published:** 2022-09-29

**Authors:** Chen-xi Li, Weihong Shi, Zhong-cheng Gong, Bin Ling

**Affiliations:** 1 Attending doctor, research assistant. Department of Oral and Maxillofacial Oncology Surgery, the First Affiliated Hospital of Xinjiang Medical University, School/Hospital of Stomatology, Xinjiang Medical University, Stomatological Research Institute of Xinjiang Uygur Autonomous Region, Urumqi, China; 2 Research assistant. Department of Oral and Maxillofacial Oncology Surgery, the First Affiliated Hospital of Xinjiang Medical University, School/Hospital of Stomatology, Xinjiang Medical University, Stomatological Research Institute of Xinjiang Uygur Autonomous Region, Urumqi, China; 3 Professor. Department of Oral and Maxillofacial Oncology Surgery, the First Affiliated Hospital of Xinjiang Medical University, School/Hospital of Stomatology, Xinjiang Medical University, Stomatological Research Institute of Xinjiang Uygur Autonomous Region, Urumqi, China; 4 Associate professor. Department of Oral and Maxillofacial Oncology Surgery, the First Affiliated Hospital of Xinjiang Medical University, School/Hospital of Stomatology, Xinjiang Medical University, Stomatological Research Institute of Xinjiang Uygur Autonomous Region, Urumqi, China

## Abstract

**Background:**

Oromaxillofacial carcinomas frequently result in serious tissue defect due to enlarged resection for treating their extensive invasion, which require challenging reconstruction. Three-dimensional (3D) printing is an advanced technology which has greatly promoted the progress of craniomaxillofacial reconstructive surgery. This present study aimed to investigate the advantages of anterolateral thigh (ALT) perforator flap manufactured by 3D printing fixed positioning guide template in curing oromaxillofacial defect.

**Material and Methods:**

Twenty patients with oromaxillofacial defects resulted from severe primary malignant tumors were divided into experimental group assisted by digital technique (n=8) and controlled group conventionally aided by ultrasound (n=12). The therapeutic effectiveness, flap preparation time, amount of bleeding, deviation of perforator vessel location, aesthetic satisfaction of donor site, postoperative complications, adverse symptom of flap, and LEFS scores were compared.

**Results:**

For experimental group, flap preparation time was significantly shorter; and it has obviously less bleeding, minor deviation of perforator vessel location, and better aesthetic satisfaction of donor site (*P*<.001). There was no statistical difference in postoperative complications and LEFS scores between two groups (*P*>.05).

**Conclusions:**

The study suggests 3D printing template of fixed positioning guide provides a brand-new method for orienting perforated vessels of ALT flap, which is more accurate in clinical application. It can improve the operative efficacy, and increase the successful rate of operation as well.

** Key words:**Anterolateral thigh perforator flap, three-dimensional printing, fixed positioning guide template, computed tomography angiography, reconstructive functional surgery.

## Introduction

Primary malignant tumors occurred in oral and maxillofacial region, with a rising incidence rate during the recent decades, are the most common in head and neck area which seriously threaten the health of patients because of their high invasion and poor prognosis (1). Given the multidisciplinary comprehensive treatment takes surgery as the principal thing, how to ensure a relatively perfect remodeling of oromaxillofacial defects whilst remove the lesion completely, is becoming more and more important. Nonetheless, that the subunits of complex anatomical structure and various physiological functions in oral and maxillofacial region, make reconstruction a difficult work. Since the anterolateral thigh (ALT) perforator flap was described by Song *et al* (2) in the year of 1984 for the first time, it has obtained a widespread popularity in recent years, especially for the reconstruction of oral and maxillofacial defects due to its multitudinous advantages (3,4). Owing to that the classical ALT perforator flap, incorporating different soft tissue components which include skin, fat, fascia, muscle, blood vessel and nerve, is pedicled with the descending branch of the lateral circumflex femoral artery (LCFA), diverse oral and maxillofacial defects could be reconstructed (5-8). What is the crux of the matter, however, is to accurately find out appropriate perforating branches of ALT flap.

Nowadays, digital medical technology and computer-aided medical procedure are increasingly being applied in the field of craniomaxillofacial reconstruction surgery, particularly the application of digital design combined with three-dimensional printing (9). Based on this novel technique, we proposed this computed tomography angiography (CTA)-aided 3D printing template to the reconstruction of defects of oral and maxillofacial region, so as to support technically in personalized and precise repair with septocutaneous artery flap.

## Material and Methods

- Clinical relevance

The digital techniques could significantly influence the surgical treatment of cancer patients, and the evidence provided in this paper may serve clinicians and dictate the next decade of personalized oral healthcare.

- Study design

All patients were recruited from the Outpatient of Oncological Department of Oral and Maxillofacial Surgery, Xinjiang Medical University Affiliated First Hospital, China, between January 2019 and December 2020. The protocol of study was approved by the Ethics Committee, Stomatological School of Xinjiang Medical University, Xinjiang Medical University Affiliated First Hospital (approval No. K202107-08), and followed the principles outlined in the Declaration of Helsinki. Informed consents were signed by all their families. All data generated or analyzed during this study are included in this published article.

The inclusion criteria were as followed: a) patients, who suffered from oral-maxillofacial soft tissue defects on account of malignant tumors, had not accepted any previous treatment; b) the general condition was accepTable for radical reconstruction operation under general anesthesia; c) skin appearance of donor-site was normal, and without cicatricial malformation, tissue disFiguration, inflammation, and any other factors that affect flap survival; d) patients were willing to receive relevant digital medical strategy.

Patients who met any of the following criteria should be excluded: a) patients with obvious organ dysfunction or organ failure; b) donor site had congenital vascular anomalies; c) patients had incomplete clinical data, and lost follow-up.

- Preoperative preparation

Processing of the CTA imaging data (experimental group only): All cases of the experimental group underwent a CTA scan ranging from anterior superior iliac spine to ipsilateral sole, keeping a supine position that sagittal plane of the body coincided with the center positioning line as well as horizontal line was located in the middle of lower extremity level. Their images were evidenced in the CTA scanner (GE Healthcare, Amersham, Buckinghamshire, UK) equipped with surface coil for helical scanning (technical parameters: tube voltage-100 kilovolt peak (kVp); effective tube current-250~300 mA; pitch of screws-0.984:1; speed of revolution-0.8~0.9 sec/turn; noise coefficient-7~8; layer thickness of scanning process-5 mm; reconstructed slice thickness-0.625 mm; reconstructive interval-0.4 mm; detector combination-64 × 0.625 mm; algebraic reconstruction technique (ART)-ASiR50%~60%; scan field of view (SFOV)-LargeBody). Moreover, the monitoring plane was at the level of renal artery; the triggering threshold value was 200 HU. A binocular high-pressure syringe (with needle head (18-20 G)) was used to inject contrast agents (iodine concentration: 350~400 mg/mL; dosage: 85~95 mL; injection rate: 4.5~5 mL/s) into anterior or median cubital vein of right arm (better than the left) applying bolus chasing methodology. Scanning delayed 10 seconds after reaching the triggering threshold, for the sequence of Runoff CTA 1.25mm SmartPrep, the total acquisition time was 5~10 min.

**Figure 1:**
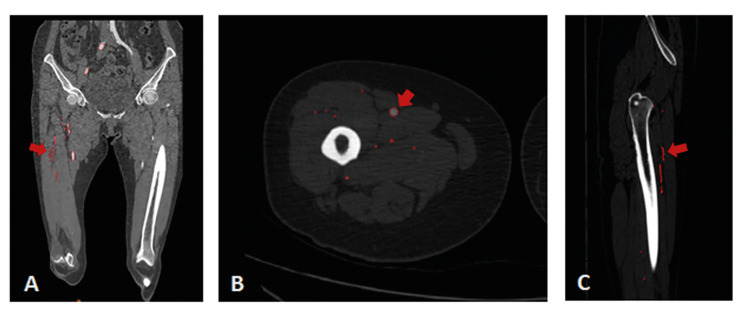
The red arrows in CTA imaging data refer to the perforator vessel of ALT flap located preoperatively (A. coronal plane; B. horizontal plane; C. sagittal plane).

CTA data in DICOM format were processed using Mimics software version 19.0 (Materialise Inc., Leuven, Belgium) to reconstruct the 3D models of skin, muscle, bone, and perforator of descending branch of LCFA and calculate its relevant information. GE AW Basic Display workstation output the CTA images (Fig. 1).

**Figure 2:**
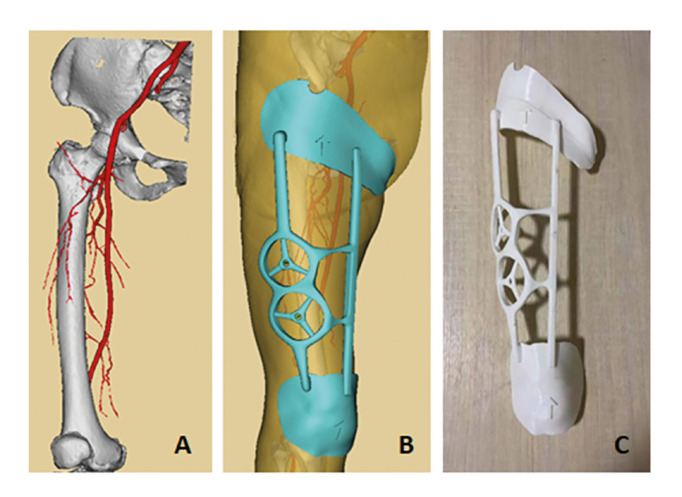
Preoperative digital design and 3D-printing fabrication of fixed positioning guide. A. osseous tissue and perforator vessels were reconstructed in line with CTA images; B. based on the previous results of reconstruction, the 3D printing template was designed; C. The 3D-printing fabrication of fixed positioning guide was completely shaped up.

Digital design and 3D-printing fabrication of fixed positioning guide (experimental group only): The concrete steps of fabricating 3D template include: a) measured 3D data were input into Geomagic Wrap 2017 (Raindrop 3D Systems Inc., USA) to be processed preliminarily to detect the incorrect plane. b) the rudiment of the model of surgical guide of lower extremity was built by using 3-matic version 9.0 (Materialise Inc., Leuven, Belgium); c) the slicing surface and plane of surgical guide were co-registered and designed accurately and precisely by Maya 2020 (Autodesk Inc., USA); d) ZBrush 2018 (Pixologic Inc., Los Angeles, California, USA) was used to smooth the bonding surface of the guide; e) after data consolidation, eventually, the digital design of surgical guide was completed via Boolean calculation; f) these integrated information, which were imported into Cura software version 15.06 (Ultimaker Inc., Utrecht, Netherlands) for coordinate axis homing, were pre-processed and provided to 3D printer in STL format; g) the proposed 3D-printing fixed positioning surgical guide of lower limb was made by fused deposition modeling (FDM) method (Fig. 2).

Conventionally ultrasonographic method for marking the position of the supplying vessels on the body surface (controlled group only): Through literature retrieval (10-12), we stipulated the regulation about positioning the descending branch of LCFA: perforator mainly originates from a circle within 6 cm of semidiameter that the center is 1~2 cm away from the midpoint of the line between the anterior superior iliac spine and the lateral edge of the patella. The average number was 3.5, which goes directly into cutaneous tissue penetrated through vastus lateralis and fascia lata.

- Surgical procedures

**Figure 3:**
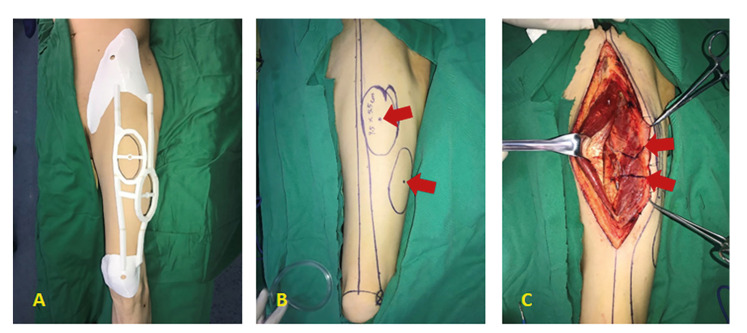
The ALT perforator flap was made by applying 3D-printing template of fixed positioning guide during operation. A. try on the fully designed 3D-printing template before operation; B. mark the skin, red arrows indicate the location of the perforator vessels; C. the perforator vessels were dissected according to the position line (pointed out by red arrows).

All patients received general anaesthesia with nasal intubation, placing in supine position and keeping buttock of operative side being padded with soft silica gel for 30 degrees, so as to better expose the operation area. The sequence of flap harvesting was as follows (take the experimental group as an example): a) surgery delineation was signed fully in consistent with 3D-printing fabrication of fixed positioning guide; b) the skin, subcutaneous tissue and fascia lata were cut successively along the medial edge of the guide template, and then separated bluntly on the surface of rectus femoris; c) after exposing the muscular septum, look for the perforating vessels to the outside; d) to judge whether the pulsating condition is good, and the direction of blood vessels is within the scope that conformed with the designed flap; e) the differences between intraoperative and preoperative location of perforating vessels were compared and analyzed as well as measured the distance between them, when the reliable perforator was found; f) open the muscular septum between rectus femoris and lateral rectus femoris along the outboard anadesma of rectus femoris, and look for the descending branch of LCFA; g) when the vascular pedicle was dissected, the lateral edge of the skin flap was cut directly to form a skin island; h) the perforating vessels were dissected continuously within the lateral femoral muscle, and the muscle sleeve tissue (about 1cm) around the perforating vessels was reserved; i) finished the amputation of pedicle, guaranteed sufficient hemostasis of donor site, the wound was inserted with negative pressure drainage tube, and sutured layer by layer; j) the divided ALT flap was transplanted to the recipient site, and fixed it at first; k) under the microscope, the adventitia of blood vessels was pruned; the blood vessels were anastomosed; the arterial pulsation was observed; the patency of blood vessels was examined by Perthes' test; and the flap was fixed by intermittent suture in the end (Fig. 3).

- Evaluation of clinical efficacy and safety

We separately recorded the preparation time of ALT perforator flap (min) and average amount of bleeding in operation (mL).

General maxillofacial examinations included scrutiny for postoperative complications comprising of bleeding, infection, vascular crisis and flap necrosis.

The aesthetic satisfaction of donor site was estimated by visual analog scale (VAS) at the onset, during and in the end of follow-up. VAS scores ranging from 0 to 10, were assessed outcome measures. Therapeutic response was graded into three levels: class I, dissatisfied (scores ranging from 0-3); class II, basically satisfied (scores ranging from 4-7); class III, immensely satisfied (scores ranging from 8-10). Classes II and III were regarded as effective cases.

Half a year after surgery, all 20 patients were evaluated according to lower extremity functional scale (LEFS) (13).

- Statistical analysis

Statistical analysis of all data was performed by Statistical Package for Natural Science (IBM SPSS version 24.0, New York, USA) and D′Agostino,R.B. test was done for normality test of measurement data. Measurement data expressed as mean ± standard deviation (SD) and values between the two groups were compared with independent sample t test, if tallied with normal distribution; or, otherwise, were calculated by inter quartile range (IQR) for Mann-Whitney U test. As for categorical data, Chi-square test or Fisher probabilities were used. *P* < 0.05 was considered statistically significant. GraphPad Prism software version 6.0 (Graph Pad Software Inc., San Diego, California, USA) was performed for plotting values.

## Results

- Basic data

This was a retrospective, observational clinical study involving 20 patients. In the light of different methods of flap preparation, they were subdivided into experimental group (n=8), applying customized 3D-printing fabrication of fixed positioning guide; and controlled group (n=12), relying on conventional ultrasonography. Among them, 7 males and 1 female with a mean age of 50.13±6.12 years old in experimental group; 10 males and 2 females with a mean age of 55.17±12.56 years old in controlled group. There was no significant difference in age (*P*=0.308), gender (*P*=0.798), and etiology between the two groups. Likewise, the similar situation happened to TNM stage (*P*=0.718), lesion position (*P*=0.413), tumor size (*P*=0.140), adjuvant therapy (*P*=0.161) (Table 1).

- Clinical efficacy and safety

The mean operative time for flap preparation of experimental group was obviously shorter (*P*<.001). The average volume of blood loss during surgery in experimental group was also conspicuously less than that of controlled group (*P*<.001).

Aside from one case appeared postoperative complications (*P*=0.402), the aesthetic satisfaction of donor site was much better with a higher VAS score in experimental group than that in controlled group (8.25±0.70 versus 6.00±1.04, *P*<.001).

Although the LEFS score of experimental group was slightly higher than that of controlled group (70.08±2.77 versus 69.88±3.31), there was no significant difference between them (*P*=0.881) (Table 2).

**Table 1:**
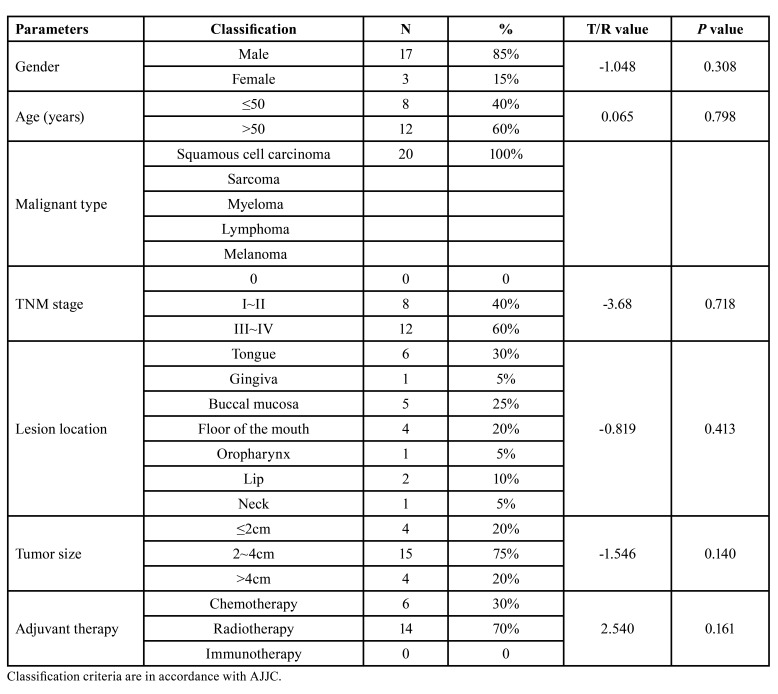
Clinical baselined information of patients.

**Table 2:**
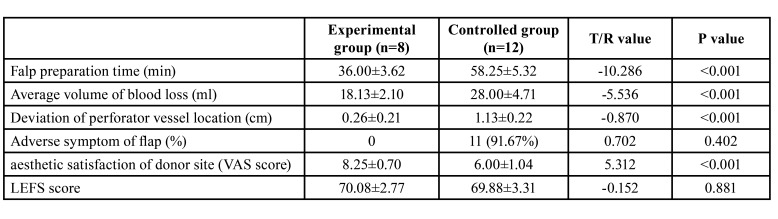
Comparison of the observational indexes between the two groups.

## Discussion

This study chiefly introduces a kind of digital medical technology for the preparation of ALT perforator flap to repair complex defect resulted from malignant tumors in maxillofacial region. Two principal innovative points have emerged from the digital design and 3D-printing fabrication. The first is that maxillofacial soft tissue defect extending to labial component can be reconstructed simultaneously, regardless of the size of the junctional defect of orolabial structure (14-16). The second is the application of 3D-printing technique, which easily facilitates the fabrication of fixed positioning guide, for ALT perforator flap preparation in a much faster and simpler way.

Traditionally, before 3D concept got into maxillofacial surgery, there were two common methods assisted to obtain ALP flap using: ultrasonography and CTA alone. Since this flap first proposed until the beginning of the new millennium, its perforators were mainly located by ultrasound; however, some cases demonstrated a vessel was used that had not been preoperatively marked and Doppler examination was unreliable and might not be readily available in identifying the perforators during planning ALT flap (2,17-19). Soon afterwards, CTA as another imaging modality, is gradually replacing ultrasound although it has purportedly removed the interobserver error associated with Doppler ultrasonography (20). In comparison to the conventional strategy, the application of the combined 3D-printing fabrication of fixed positioning guide introduced in the current study, is characterized by additional benefits including a more precise personalized perforator mapping, a significantly shortened surgical time, and a greatly reduced intraoperative bleeding volume. Furthermore, the reconstruction is much more accurate and sTable than which achieved with ALT flap graft prepared by ultrasound due to the use of the patient-fitted design principal.

On the other hand, the customized 3D-printing fabrication of fixed positioning guide for ALT perforator flap harvest was developed based on advanced 3D medical image scanning and processing software. It can commendably visualize the deep source artery as well as its perforators. Owing to this crucial point, we could diminish the intraoperative trauma for flap dissection, hence, not only did we raise the accuracy and efficacy of reconstructing complex defects, but also the donor-site morbidity was none and with higher postoperative LEFS score. And that agreed with Battaglia *et al* (21) who utilized free vascularized fibular myocutaneous flap to reconstruct mandibular defects. We also found that experimental group’s variation of perforator location was remarkably less than the controlled (0.26±0.21 versus 1.13±0.22, *P*<.001), in other words, the mapping accuracy of the experimental group was relatively high. In addition, importantly, these results predicted in our system was in accordance with an intraoperative investigation of the actual perforators depending on anatomic landmarks (22), therefore, it avoided the error of positioning and reduced the risk of injury of perforator vessels concurrently.

In conclusion, CTA combined with customized 3D-printing fabrication of fixed positioning guide is a precise and reliable protocol for anticipating the perforators of ALT flap, and it can be safely dissected as well. It also represents a new trend in craniomaxillofacial reconstructive surgery with the application of combinations of digital medicine, 3D-printing technology, bioengineering and material science to aid in those complicated and challenging lesions. So we conclude that utilizing this system is one approach in consistently dissecting the ALP flap.

## References

[1] Chi AC, Day TA, Neville BW (2015). Oral cavity and oropharyngeal squamous cell carcinoma--an update. CA Cancer J Clin.

[2] Song YG, Chen GZ, Song YL (1984). The free thigh flap: a new free flap concept based on the septocutaneous artery. Br J Plast Surg.

[3] Gong ZJ, Zhang S, Wang K, Tan HY, Zhu ZF, Liu JB (2015). Chimeric flaps pedicled with the lateral circumflex femoral artery for individualised reconstruction of through-and-through oral and maxillofacial defects. Br J Oral Maxillofac Surg.

[4] Gong ZJ, Ren ZH, Wang K, Tan HY, Zhang S, Wu HJ (2017). Reconstruction design before tumour resection: A new concept of through-and-through cheek defect reconstruction. Oral Oncol.

[5] Gong ZJ, Wu HJ (2013). Measurement for subcutaneous fat and clinical applied anatomic studies on perforators in the anterior thigh region. J Oral Maxillofac Surg.

[6] Chen YC, Scaglioni MF, Carrillo Jimenez LE, Yang JC, Huang EY, Lin TS (2016). Suprafascial Anterolateral Thigh Flap Harvest: A Better Way to Minimize Donor-Site Morbidity in Head and Neck Reconstruction. Plast Reconstr Surg.

[7] Wei FC, Jain V, Celik N, Chen HC, Chuang DC, Lin CH (2002). Have we found an ideal soft-tissue flap? An experience with 672 anterolateral thigh flaps. Plast Reconstr Surg.

[8] Yu P (2004). Characteristics of the anterolateral thigh flap in a Western population and its application in head and neck reconstruction. Head Neck.

[9] Meglioli M, Naveau A, Macaluso GM, Catros S (2020). 3D printed bone models in oral and cranio-maxillofacial surgery: a systematic review. 3D Print Med. 2020;6:30. Erratum in: 3D Print Med.

[10] Zhou H, Tan Q, Liu S, Zhou H, Wang S, Yan X (2013). Improved location technology of perforators of anterolateral thigh flap for Chinese patients. J Reconstr Microsurg.

[11] Smith RK, Wykes J, Martin DT, Niles N (2017). Perforator variability in the anterolateral thigh free flap: a systematic review. Surg Radiol Anat.

[12] Yu P, Selber J, Liu J (2013). Reciprocal dominance of the anterolateral and anteromedial thigh flap perforator anatomy. Ann Plast Surg.

[13] Binkley JM, Stratford PW, Lott SA, Riddle DL (1999). The Lower Extremity Functional Scale (LEFS): scale development, measurement properties, and clinical application. North American Orthopaedic Rehabilitation Research Network. Phys Ther.

[14] Tischendorf L, Bernt H, Fröhlich M, Gitt HA, Rink B, Seela W (1989). Multicentric retrospective study on the carcinoma of lips and oral mucosa. Analysis of coherence between pre- and posttherapeutical categories of classification. Stomatol DDR.

[15] Zitsch RP 3rd (1993). Carcinoma of the lip. Otolaryngol Clin North Am.

[16] Sasaki K, Sasaki M, Oshima J, Aihara Y, Nishijima A, Sekido M (2020). Free-flap reconstruction for full-thickness oral defects involving the oral commissure combined with oral modiolus reconstruction using a fascial sling. Microsurgery.

[17] Giunta RE, Geisweid A, Feller AM (2000). The value of preoperative Doppler sonography for planning free perforator flaps. Plast Reconstr Surg.

[18] Tsukino A, Kurachi K, Inamiya T, Tanigaki T (2004). Preoperative color Doppler assessment in planning of anterolateral thigh flaps. Plast Reconstr Surg.

[19] Hallock GG (2003). Doppler sonography and color duplex imaging for planning a perforator flap. Clin Plast Surg.

[20] Rozen WM, Phillips TJ, Ashton MW, Stella DL, Gibson RN, Taylor GI (2008). Preoperative imaging for DIEA perforator flaps: a comparative study of computed tomographic angiography and Doppler ultrasound. Plast Reconstr Surg.

[21] Battaglia S, Maiolo V, Savastio G, Zompatori M, Contedini F, Antoniazzi E (2017). Osteomyocutaneous fibular flap harvesting: Computer-assisted planning of perforator vessels using Computed Tomographic Angiography scan and cutting guide. J Craniomaxillofac Surg.

[22] Lin SJ, Rabie A, Yu P (2010). Designing the anterolateral thigh flap without preoperative Doppler or imaging. J Reconstr Microsurg.

